# Isoform of APOE with retained intron 3; quantitation and identification of an associated single nucleotide polymorphism

**DOI:** 10.1186/1750-1326-5-34

**Published:** 2010-09-07

**Authors:** Laura S Dieter, Steven Estus

**Affiliations:** 1 Department of Physiology and Sanders-Brown Center on Aging, University of Kentucky, Lexington, KY, USA

## Abstract

**Background:**

Alleles of apolipoprotein E (*APOE*) are the major genetic risk factor for late onset Alzheimer's Disease (LOAD). Recently, an *APOE *splice variant that retains intron 3 (*APOE-I3*) was identified. To gain insight into the possible role of this isoform in LOAD, we quantified its expression in a cohort of 56 human brain specimens by using quantitative RT-PCR.

**Results:**

We found that *APOE-I3 *generally represents a low percentage (< 0.5%) of overall *APOE *expression. However, in one specimen, the proportion of *APOE-I3 *was increased about ~13 fold. This specimen was unique in the cohort for possessing the minor allele of an intron 3 single nucleotide polymorphism (SNP), rs12982192. Additionally, an allelic expression imbalance study indicated that the rs12982192 minor allele was associated with increased *APOE-I3 *expression.

**Conclusions:**

Overall, we interpret our results as suggesting that *APOE-I3 *represents a minor portion of *APOE *expression and that rs12982192 is associated with *APOE *intron 3 retention. Since the minor allele of this SNP is on the same haplotype as the minor allele of rs429358, which defines the *APOE*4 allele, we speculate that rs12982192 may reflect a modest loss of mRNA encoding functional *APOE*4.

## Background

*APOE *variants are the single largest genetic factors impacting late onset Alzheimer Disease (LOAD), the most common neurodegenerative disease. In particular, the *APOE*4 allele confers increased risk, being present at a ~15% frequency in the general population and at 50% in LOAD [1,2]. *APOE *consists of 4 exons and 3 introns encoding a total of 317 amino acids. After cleavage of the signal peptide, the mature APOE protein is 299 amino acids. Protein translation begins in exon 2 and ends in exon 4. In the brain, APOE transports cholesterol and modulates Aß clearance [1,2]. APOE is primarily secreted by astrocytes [3,4] with microglia [5] and, possibly, stressed neurons also contributing to APOE production [6]. Overall, insights into *APOE *expression will provide further information on AD risk.

Recently, Xu et al reported an *APOE *isoform that retained intron 3 (*APOE-I3*) and was increased with neuronal stress in murine models [7]. To gain insights into this novel *APOE *isoform in humans, we quantified *APOE-I3 *as well as the normal length *APOE *(*APOE-NL*) in a cohort of 56 AD and non-AD brain samples. We report that *APOE-I3 *represents a low proportion of overall *APOE *expression. In addition, we identified an individual with significantly increased expression of the *APOE-I3 *isoform. Further analysis revealed that this individual was unique in the cohort for an intron 3 single nucleotide polymorphism (SNP), rs12982192. Moreover, *APOE-I3 *was in allelic expression imbalance (AEI) in this individual such that the minor allele of rs12982192 correlated with increased expression of the *APOE-I3 *isoform. Overall, we interpret our results as suggesting that *APOE-I3 *represents a minor portion of *APOE *expression and that rs12982192 is associated with *APOE *intron 3 retention.

## Materials and methods

### Human autopsy tissue

The human autopsy tissue specimens used here have been described elsewhere [8,9]. Briefly, anterior cingulate tissue was provided by the University of Kentucky AD Center. Diagnoses of AD and non-AD were based upon evaluation of both cognitive status, i.e., Clinical Dementia Rating and Mini-Mental State Examination scores, as well as neuropathology [10]. The age at death for individuals that were cognitively intact, i.e., non-AD, was 82 ± 9 years (mean ± SD, n = 29) while age at death for AD individuals was 82 ± 6 (n = 27). The average post-mortem interval for non-AD and AD individuals was 2.8 ± 0.8 hours (mean ± SD, n = 29) and 3.4 ± 0.6 hours (n = 27), respectively. The non-AD and AD individuals were well-balanced for sex, i.e., 15 of the 29 non-AD individuals were women while 14 of the 27 AD individuals were women.

### PCR amplification

**Figure 1:**
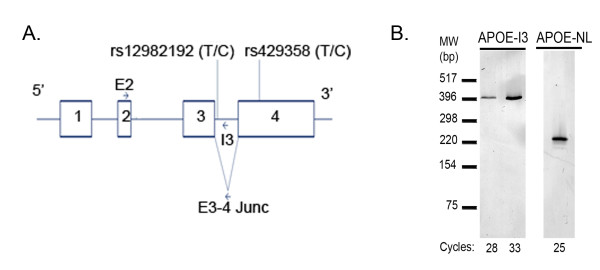
**PCR Detection of *APOE-NL *and *APOE-I3***. PCR primer positions are depicted relative to the four exons of *APOE *(A). Two SNPs are also shown: rs12982192 within intron 3 and rs429358, which determines *APOE*4 status, in exon 4. PCR of 25 or 28-33 cycles was used to detect *APOE-NL *and *APOE-I3*, respectively, in pooled brain cDNA samples (B).

RNA was extracted and converted to cDNA as described previously [8,9]. To initially visualize the *APOE *isoforms (Figure 1A), *APOE-I3 *was amplified with primers that corresponded to sequences within exon 2 (primer E2: CGTTGCTGGTCACATTCCT) and intron 3 (I3: AGAGAGAGACAAAGCTGAGA). The normal length *APOE *isoform (*APOE-NL*) was amplified with the same exon 2 primer and a reverse primer that corresponded to the exon 3-4 junction (E3-4 Junc: CCATCAGCGCCCTCAGTT) (Figure 1A). The equivalent of 20 ng of template cDNA was amplified with 1 μM of each primer in a PCR profile consisting of pre-incubation for 3 minutes at 94°C, followed by cycles of 94°C for 30 sec, 60°C for 30 sec and 72°C for 30 sec (Perkin Elmer 9600). PCR products were separated by polyacrylamide gel electrophoresis and visualized by SYBR-gold staining and fluorescence imaging (FujiFLA-2000). The PCR products were isolated from the gel and identified by direct sequencing (Davis Sequencing). For real-time quantitative PCR (RT-qPCR), reaction mixtures consisting of 1 uM each primer, 1× SYBR-green Master Mix (Applied Biosystems) and 20 ng of template cDNA were pre-incubated for 10 minutes at 95°C followed by 40 cycles of 94°C for 30 sec, 60°C for 30 sec and 72°C for 30 sec (BioRad Chromo4). After completion of the RT-qPCR cycles, assay specificity was confirmed by melting curve analysis and by visualizing the products by polyacrylamide gel electrophoresis and SYBR-gold staining. Copy numbers were determined relative to standard curves that were amplified in parallel; the standards were generated from PCR-amplified products that were gel purified and quantified by UV absorbance. Each sample was analyzed twice by RT-qPCR. The limit of detection was five copies of *APOE *isoform per 20 ng of cDNA. Copy numbers were normalized to the geometric mean of the copy numbers of hypoxanthine-guanine phosphoribosyltransferase 1 and ribosomal protein L32 [11,12]. Samples were genotyped for rs12982192 by using a TaqMan approach (Applied Biosystems).

### Allelic expression imbalance

To determine whether each allele of rs12982192 and rs429358 contributed equally to *APOE-I3 *and *APOE-NL*, respectively, we cloned the *APOE-I3 *and *APOE-NL *PCR products from specimen #31 (TA-Cloning pcDNA3.1, Invitrogen). The identity of each clone was confirmed by direct sequencing (Davis Sequencing). We then estimated the proportion of each allele present in specimen #31 *APOE-I3 *and *APOE-NL *cDNA by comparing the relative sequencing electropherogram peak areas of each allele of rs12982192 and rs429358, respectively, essentially as described [13]; the results of this assay were normalized relative to standard curves derived from varying proportions of each allelic clone and validated by analysis of genomic DNA (gDNA).

### Statistical Analysis

The association between *APOE-I3 *and/or *APOE-NL *and sex, age, PMI and *APOE*4 genotype was analyzed by linear regression (SPSS v 17.0). Differences in AEI in gDNA and cDNA were evaluated by a two-tailed T-test (SPSS, V17). A p < 0.05 was considered to be significant.

## Results

**Figure 2:**
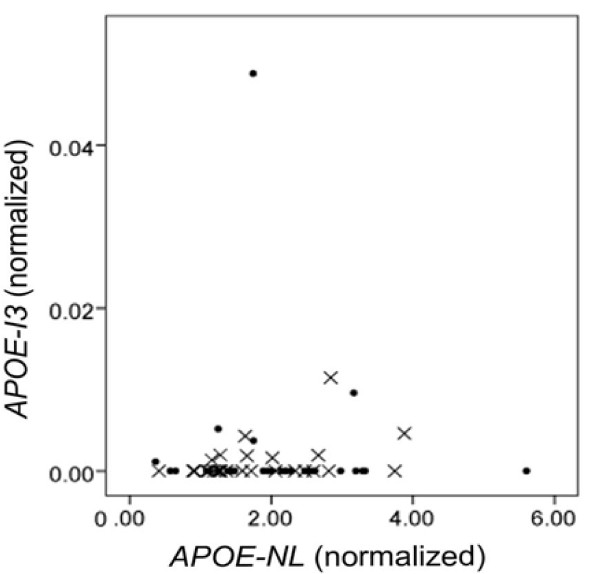
**Quantification of *APOE-I3 *and *APOE-NL *Expression**. Expression of *APOE-I3 *is shown as a function of *APOE-NL *in AD (X markers) and non-AD (circle markers) samples. Specimen #31 is the non-AD outlier with a high level of *APOE-I3 *expression. RT-qPCR data were normalized to the geometric mean of hypoxanthine-guanine phosphoribosyltransferase and ribosomal protein L32. There was no correlation of *APOE-I3 *or *APOE-NL *with *APOE*3 versus *APOE*4 genotype, or AD status (data not shown).

To begin to evaluate *APOE-I3 *and *APOE-NL *expression, we amplified human brain cDNA by using a forward primer within exon 2 (E2) and reverse primers within intron 3 (I3) or the junction of exon 3-4 (E3-4 Junc), respectively (Figure 1A). Both the *APOE-I3 *and the *APOE-NL *isoform were readily discernible (Figure 1B). We then used RT-qPCR to quantify each *APOE *isoform in 20 ng of cDNA prepared from 56 human brain samples and the results normalized for housekeeping gene expression. We found that *APOE-I3 *expression was low relative to *APOE-NL *expression (Figure 2). Indeed, the *APOE-I3 *isoform was not detected in 43 of the 56 specimens and was very low in another 12 of the samples, averaging 0.22% of *APOE *message. Interestingly, the cDNA from the remaining sample (specimen #31) had much higher *APOE-I3*, reaching 2.8% of total *APOE *expression (Figure 2). This autopsy brain specimen was from a 91 year old, cognitively-intact person with a Braak stage of II and an *APOE*3*/*4 genotype. Excluding this outlier and samples where *APOE-I3 *was not detected, there was a strong trend between the expression of *APOE-NL *and *APOE-I3 *(p = 0.053). The expression of neither isoform correlated with AD diagnosis, PMI, gender, *APOE *genotype or age (p > 0.05). Hence, *APOE-I3 *is a relatively rare *APOE *isoform in this cohort, with one specimen showing ~13-fold higher *APOE-I3 *levels.

To identify SNPs that may underlie the increased *APOE-I3 *in specimen #31, we generated genomic *APOE *clones from exon 2 to exon 4 of this individual. These clones included the intervening 1,092 bp intron 2 and 580 bp intron 3. Sequencing revealed that this gDNA was heterozygous for (i) rs12982192, which is a rare T/C SNP located 50 bp into intron 3 (dbSNP Build 131), (ii) rs769449, which has a 6.5% minor allele frequency and is located 78 bp into intron 2, (iii) rs769450, which has a 39.9% minor allele frequency and is also located within intron 2, and (iv) rs429358, which is within exon 4 and determines *APOE*3/4 status (Figure 1A). The *APOE*4 haplotype (rs429358C) contained the minor rs12982192C and rs769449A alleles and the major rs769450G allele. Since rs12982192 is within the retained intron 3, we hypothesized that the minor rs12982192C allele may be associated with *APOE-I3*. To test this possibility, we first assessed whether the minor allele of rs12982192 was present in any of the other 55 DNA samples in this study; only specimen #31 possessed the minor rs12982192C allele. This finding supports the possibility that intron 3 retention is associated with rs12982192.

**Figure 3:**
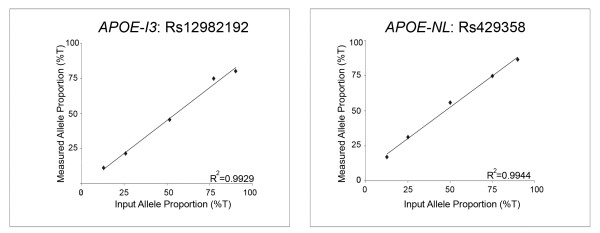
**AEI Standard Curves**. These standard curves were generated by subjecting the indicated proportions of allelic clones to AEI analysis. The resulting quantified allelic proportions were linear with respect to input proportions as noted by the indicated correlation coefficients. The slopes of the lines for rs12982192 and rs429358 were 0.94 and 0.88, respectively.

**Figure 4:**
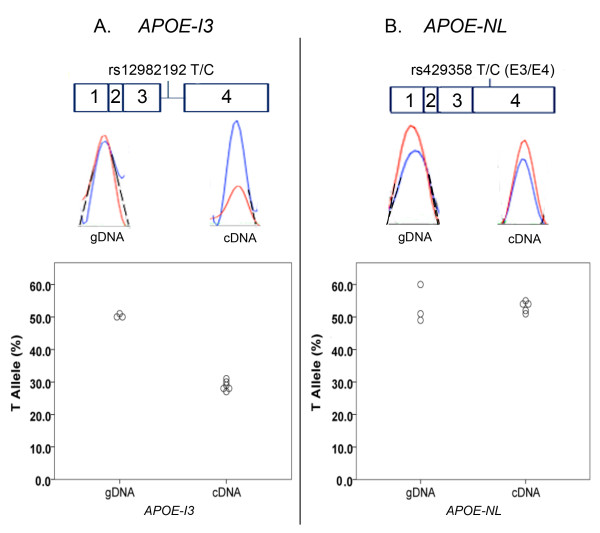
***APOE-I3 *but not *APOE-NL *shows AEI in Specimen #31**. The upper portion shows each *APOE *isoform and the placement of the SNP used to evaluate its AEI. The middle portion shows representative electropherograms for assessing AEI in *APOE-I3 *(A) and *APOE-NL *(B). The lower portion represents quantitative results for genomic DNA, which shows equal representation of each allele, and for cDNA, which shows unequal allelic expression for *APOE-I3 *but not *APOE-NL*. Electropherograms were analyzed for AEI by extrapolating the curves to baseline (dashed lines) and then estimating relative allelic areas. These data were normalized relative to standard curves generated from mixtures of allelic clones (Figure 3).

To evaluate this possibility further, we reasoned that *APOE-I3 *should be in allelic expression imbalance (AEI) with respect to rs12982192 genotype, that is, we hypothesized that the minor rs12982192C allele would be over-represented in *APOE-I3*. Hence, we compared the proportion of each rs12982192 allele in *APOE-I3*. In addition, to evaluate whether the bulk of *APOE *expression, as reflected by *APOE-NL*, was in AEI in specimen #31, we also analyzed *APOE-NL *by using rs429358 in exon 4. Standard curves demonstrated the linearity of the AEI assay (Figure 3). The assay was further validated by testing gDNA, which found that, as expected, each allele was present in equal proportions for both rs12982192 and rs429358 (Figure 4A-B). As previously reported for *APOE *expression generally [14], *APOE-NL *expression in specimen #31 did not show evidence of AEI (Figure 4B). In contrast, *APOE-I3 *showed robust AEI in specimen #31, with significant over-representation of the minor rs12982192C allele (Figure 4A). Hence, these results support the hypothesis that the minor rs12982192C allele is associated with increased *APOE-I3*.

## Discussion

The major findings of this paper are (i) *APOE-I3 *appears to be a rare *APOE *isoform compared to *APOE-NL *in the aged human brain, (ii) *APOE-I3 *was increased ~13-fold in one individual, (iii) rs12982192, an intron 3 SNP, was uniquely present in the specimen with high *APOE-I3*, and (iv) *APOE-I3 *expression showed significant AEI in this sample, with the minor rs12982192 allele contributing a greater proportion of *APOE-I3 *expression. Hence, rs12982192 appears to be associated with increased expression of a rare *APOE *isoform.

Although the role of apoE-I3 in the brain is unclear, this isoform is infrequent relative to *APOE-NL*. While *APOE *is expressed largely in astrocytes [3,4,15], Xu et al found that *APOE-I3 *expression was restricted to neurons in mice [7]. We did not discern a correlation between the expression of *APOE-I3 *and synaptophysin, an mRNA restricted to neurons, although this correlation may have been precluded by the generally low *APOE-I3 *expression such that only 13 of the 56 samples with detectable *APOE-I3 *contributed to this analysis. While *APOE-NL *encodes a 317 amino acid secreted protein, the sequence at the exon 3 - intron 3 junction results in a stop codon. Hence, *APOE-I3 *encodes a truncated 79 amino acid, amino-terminal APOE fragment that is apparently not detectable in transfected cells or brain [7]. Although we considered that *APOE-I3 *may be a candidate for nonsense-mediated decay, *APOE-I3 *levels were independent of PMI (data not shown), suggesting that *APOE-I3 *is not rapidly degraded. In summary, the role of *APOE-I3 *in the brain is unclear but, given that this isoform is rare and that its encoded protein is not detectable, we speculate that *APOE-I3 *represents a loss of APOE mRNA encoding functional protein.

Several lines of evidence suggest that rs12982192 modulates the proportion of *APOE-I3*, including (i) rs12982192 resides within intron 3 consistent with its possible role as a *cis*-acting SNP, (ii) this SNP was uniquely present in the specimen with relatively high *APOE-I3 *among the 56 brain specimens analyzed, (iii) rs12982192 was associated with significant AEI for *APOE-I3 *with the rare rs12982192C minor allele contributing a greater proportion of *APOE-I3 *expression and (iv) the *APOE-NL *isoform did not display AEI similar to the *APOE-I3 *isoform, ruling out the possibility of an overall *APOE *AEI in specimen #31 due to other mechanisms, e.g., a promoter SNP. While this reasoning appears robust to support a role for rs12982192 in *APOE-I3 *splicing, two lines of evidence suggest that rs129382192 does not act independently to modulate intron 3 splicing. First, rs129382192 did not alter *APOE *splicing in a transfected cell, minigene paradigm (Dieter and Estus, unpublished observations), suggesting *in vivo *elements may be necessary to discern the actions of the SNP. Second, while the main finding of the AEI study was that the minor allele of rs12982192 was associated with increased *APOE-I3 *expression, a secondary finding was that the ~3.5:1 allelic ratio was quantitatively insufficient to account for the ~13-fold increase in overall *APOE-I3 *expression in specimen #31. We interpret this result as possibly reflecting additional variables specific to specimen #31 that influence *APOE-I3 *or, more likely, that the difference is attributable to currently unclear technical differences between the RT-qPCR and AEI assays. In summary, although *APOE-I3 *levels may be modulated by additional variables, several lines of evidence support the association of rs12982192 with *APOE-I3 *expression.

Since *APOE*4 is the primary genetic risk factor for AD, SNPs that alter *APOE*4 expression are likely to modulate the association of *APOE*4 with AD. In this regard, we note that the minor rs12982192C allele is on the same haplotype as the minor rs429358C allele, which defines the *APOE*4 allele. Since our AEI study indicates that rs12982192 is associated in *cis *with retention of *APOE *intron 3, rs12982192 may emerge as a modulator of *APOE*4 allelic association with AD. Hence, although *APOE-I3 *represents only ~3% of total *APOE *mRNA in the rs12982192 heterozygous individual, the AEI study indicates that the majority of this *APOE-I3 *is derived from the *APOE*4 allele, and therefore could approach 5-6% of *APOE*4 expression. The extent that rs12982192 may be associated with AD risk will require a very large study population, given the rarity of this SNP, i.e., this SNP is listed within dbSNP Build 131 without a true frequency. We note that we have genotyped an additional ~800 DNA samples and have not identified another rs12982192 carrier.

In summary, our major findings are that *APOE-I3 *is a low abundance *APOE *isoform. Moreover, the minor C allele of rs12982192 appears associated with increased *APOE-I3*. Since the minor rs12982192C allele is on the same haplotype as *APOE*4, and since *APOE-I3 *appears to represent a loss of functional *APOE*4 mRNA, rs12982192 may modulate the association of *APOE*4 with AD risk. Future large case-control association studies are necessary to evaluate this possibility.

## Abbreviations

(*APOE*): apolipoprotein E; (LOAD): late onset Alzheimer's Disease; (*APOE-I3*): *APOE *splice variant retaining intron 3; (SNP): single nucleotide polymorphism; (*APOE-NL*): normal length *APOE*; (AEI): allelic expression imbalance; (RT-qPCR): real-time quantitative PCR; (gDNA): genomic DNA.

## Competing interests

The authors declare that they have no competing interests.

## Authors' contributions

LSD performed the experiments under the supervision of SE. Both authors contributed to data analysis and writing the manuscript. Both authors read and approved the final manuscript.
